# Real-world effectiveness of psychiatric pharmacotherapies in reducing the risk of substance use- and psychiatric hospitalisations in cannabis use disorder – An exploratory nationwide register-based study

**DOI:** 10.1192/j.eurpsy.2026.12250

**Published:** 2026-08-12

**Authors:** Antti Mustonen, Solja Niemelä, Markku Lähteenvuo, Alexander Denissoff, Antti Tanskanen, Ellenor Mittendorfer-Rutz, Jari Tiihonen, Heidi Taipale

**Affiliations:** 1 Faculty of Medicine and Health Technology, Tampere University, Tampere, Finland; 2 Department of Clinical Neuroscience, Karolinska Institutet, Stockholm, Sweden; 3 Department of Psychiatry, Seinäjoki Central Hospital, Seinäjoki, Finland; 4 Department of Psychiatry, Faculty of Medicine, University of Turku, Turku, Finland; 5 Addiction Psychiatry Unit, Department of Psychiatry, Turku University Hospital, Wellbeing County of South-West Finland, Turku, Finland; 6 Department of Forensic Psychiatry, University of Eastern Finland, Niuvanniemi Hospital, Kuopio, Finland; 7 Neuroscience Center, University of Helsinki, Helsinki, Finland; 8 Center for Psychiatry Research, Stockholm Region, Stockholm, Sweden

**Keywords:** cannabis use disorder, hospital treatment, outcome, pharmacotherapy, real-world effectiveness, register

## Abstract

**Background:**

Pharmacotherapies have shown limited efficacy in cannabis use disorder (CUD). This exploratory study examined the real-world effectiveness of commonly used psychiatric medications in reducing the risk of substance use and psychiatric hospitalisations among individuals with CUD.

**Methods:**

We conducted a nationwide prospective cohort study using Swedish health and administrative registers. The cohort included 51,947 individuals aged 16–64 years with a first CUD diagnosis (ICD-10 F12) between 2006 and 2021. Medication use was modelled as time-dependent with the PRE2DUP method. Within-individual Cox regression was used to compare hospitalisation risk during medication use versus non-use. The primary outcome was hospitalisation for substance use disorder (SUD).

**Results:**

ADHD (aHR: 0.85; 95% CI: 0.81–0.88) and SUD medications (aHR: 0.97; 0.93–0.99) were associated with reduced SUD hospitalisation risk. In contrast, antipsychotics (aHR: 1.12; 1.09–1.15), antidepressants (aHR: 1.09; 1.06–1.11), and benzodiazepines (aHR: 1.14; 1.11–1.17) were associated with increased risk, while mood stabilisers showed no significant effect. Specific agents, including clozapine (aHR: 0.47; 0.31–0.73), naltrexone (aHR: 0.79; 0.71–0.88), buprenorphine (aHR: 0.92; 0.88–0.96), lisdexamfetamine (aHR: 0.74; 0.69–0.79), methylphenidate (aHR: 0.90; 0.86–0.94), and ADHD polytherapy (aHR: 0.85; 0.75–0.96), were associated with reduced risk.

**Conclusions:**

Most psychiatric medications were not associated with a reduced risk of SUD in individuals diagnosed with CUD. However, pharmacotherapies for ADHD, opioid or alcohol use disorder, and treatment-resistant schizophrenia were linked to better outcomes, likely reflecting the underlying clinical trajectories of other comorbid psychiatric disorders rather than direct therapeutic effects on CUD.

## Introduction

The prevalence of cannabis use is increasing globally [1], with several countries taking steps to decriminalise or legalise non-medical cannabis. This may result in more potent cannabis products being available at lower prices, which could lead to increased cannabis use and a corresponding rise in individuals presenting with cannabis use disorders (CUDs) [1, 2]. CUD is a potential risk associated with cannabis use. It is characterised by continued use despite significant adverse effects, a compulsive need for repeated use, a diminished ability to control use, escalating doses, the development of tolerance, and a lack of interest in other aspects of life [3]. Approximately 1 in 10 regular cannabis users develop CUD, and the rate increases to up to 1 in 3 among those who use cannabis daily [1]. Cumulatively, the increasing number of affected individuals from CUD imposes a major burden on healthcare and social insurance systems.

There are no approved pharmacotherapies for the treatment of CUD, making psychosocial treatments such as cognitive behavioural therapy and motivational interviewing first-line treatments. To date, there have been three Cochrane reviews of the effectiveness of pharmacological treatments in the treatment of CUD. These reviews have found incomplete and low to very low quality of evidence for all of the pharmacotherapies and outcomes [4–6]. The most recent extension of these studies in 2025 [6] included pharmacotherapeutic interventions such as tetrahydrocannabinol (THC) and cannabidiol (CBD) preparations, antidepressants with different mechanisms of action, mood stabilisers or anticonvulsants, and antipsychotics. The authors concluded that all studied pharmacotherapies should be considered experimental for CUD treatment given the limited evidence of efficacy. Current data therefore suggest only a minimal role for pharmacotherapy in the management of CUD.

Research indicates that cannabis use is associated with a range of adverse outcomes, including an increased risk of developing mental health disorders [7] and worsening the prognosis of existing conditions [8, 9]. The growing number of individuals seeking help for CUD [10], combined with the limited availability of psychosocial interventions [11] and the increasing burden of mental ill-health associated with cannabis use [12–14], call for interventions. Current data from randomised controlled trials (RCTs) are derived from studies with small sample sizes, and no studies have assessed long-term outcomes such as hospitalisations. Large-scale register studies with advanced methodology can fulfil these research gaps and provide crucial information on the real-world effectiveness of various pharmacological treatments while considering relevant individual factors such as work-life-related capacity. Leveraging nationwide register data covering all Swedish residents, this exploratory study aims to examine the real-world effectiveness of different pharmacotherapies in reducing the risk of substance use and psychiatric hospitalisations among individuals with CUD. This study has the potential to influence the design of future RCTs aimed at improving treatment strategies.

## Methods

### Study population

This study uses several Swedish nationwide registers covering all individuals with Swedish residency. All Swedish residents are assigned a unique personal identification number, which enables linkage between various registers. These registers include the National Patient Register (NPR), Micro Data for Analyses of Social Insurance (MiDAS) register, Cause of Death Register (CDR), Prescribed Drug Register (PDR), and Longitudinal Integration Database for Health Insurance (LISA) register. NPR includes data on in-patient and specialised out-patient care periods, while the MiDAS register includes data on sickness absence and disability pension, namely data on periods during which individuals have received sickness benefits because of health-related incapacity for work. From the NPR and MiDAS register, we identified all individuals aged 16–64 years who were recorded with a CUD diagnosis (ICD-10 code F12.x) between January 2006 and December 2023 without any exclusion criteria. Sociodemographic data (age, gender, educational level, country of birth, and occupational data) were derived from the LISA register. We used data from the REWHARD consortium that was supported by the Swedish Research Council (grant number 2021-00154). The research project was approved by the Regional Ethics Board of Stockholm, Karolinska Institutet, Stockholm, Sweden (decision 2007/762-31 and Dnr 2021-06441-02). According to current Swedish law, the use of registry data for research purposes does not require informed consent from individuals held in these registries. We used the STROBE reporting guidelines for cohort studies. The STROBE checklist is included in the Supplementary Material.

### Key variables and covariates

Sociodemographic data, including age, sex, and country of birth, were obtained from LISA. Data on disability pension status at cohort entry were obtained from MiDAS. Medication data were gathered from the PDR from July 2005 to December 2024 and were categorised into different pharmacotherapies based on Anatomical Therapeutic Chemical (ATC) classification codes [15]. These medications were categorised based on their ATC classification as medications for SUDs (N07BB and N07BC), medications for attention-deficit hyperactivity disorder (ADHD) (N06BA), mood stabilisers (N03AF01, N03AG01, N03AX09, and N05AN01), antidepressants (N06A), benzodiazepines and related drugs (N05BA, N05CD, and N05CF), and antipsychotics (N05A, excluding N05AN01 [lithium]). These categories are described in the Supplementary Material in more detail. Medication data were modelled into medication use periods (i.e., when medication use started and ended) with the PRE2DUP (from prescription drug purchases to drug use periods) method [16].

### Outcomes

The primary outcome was hospital admission related to SUD (ICD-10 code F10-F19 as main diagnosis). Secondary outcomes were (a) hospital admission related to CUD (ICD-10 code F12 as the main diagnosis) and (b) hospital admission related to any psychiatric disorder (ICD-10 codes F00–F99 as the main diagnosis).

### Statistical methods

Exposure to different pharmacotherapies was modelled in a time-dependent manner and updated in the models when any change to pharmacotherapy in use occurred (i.e., switch, add-on, and discontinuation). Each medication class (antipsychotics, antidepressants, mood stabilisers, ADHD drugs, SUD medications, and benzodiazepines) was studied separately, with the use of specific pharmacotherapy compared with non-use of that medication class (e.g., the reference category for risperidone is non-use of antipsychotics). Medication data with fewer than five events are shown as <5.

We used a within-individual design with stratified Cox regression models in which each individual formed his or her own stratum [17]. This reduces selection bias, controlling for the time-invariant factors such as baseline comorbidities and genetics. We calculated adjusted hazard ratios (aHRs) and 95% confidence intervals (CIs) comparing the risk of outcomes during time periods of use of different pharmacotherapies with time periods of non-use of these pharmacotherapies in individuals with CUD. Temporal order of treatments, time since cohort entry, and time-varying use of other psychotropic medication were adjusted for in analyses. The follow-up time was reset to zero after the outcome event, meaning that main outcomes were treated as recurrent events. Patients were followed up from CUD – diagnosis until emigration (LISA), death (CDR), or end of the data linkage (December 2024), whichever occurred first.

To bolster the validity of our findings, we conducted two sensitivity analyses for the main outcome (SUD). First, to minimise the potential impact of comorbid SUDs in baseline, we excluded individuals with SUD diagnoses within 1 year (*n* = 12,614) before cohort entry (i.e., before the first CUD diagnosis). We then applied a within-individual design using a stratified Cox regression model, consistent with our primary models. Results are presented in Supplementary Figure S1. Second, we performed between-individual Cox regression analyses that were adjusted for the temporal order of pharmacotherapies, time-varying use of other psychotropic medications, age at cohort entry, gender, disability pension status at cohort entry, number of previous hospitalisations for CUD, and records of epilepsy, diabetes, cancer, cardiovascular disorders, personality disorders, accidental overdoses, and suicide attempts (Supplementary Figure S2). Results are presented in Supplementary Figure S2. Covariate definitions are presented in Supplementary Table S1.

Statistical significance was considered as *p* < 0.05 after Benjamini–Hochberg adjustment for multiple comparisons. Statistical analyses were performed using SAS version 9.4. Forest plot figures were created using R version 4.3.1 using the forestploter package.

## Results

The cohort included 51,947 individuals with CUD, of which 39,276 (75.61%) were men, 10,277 (19.78%) were born outside Sweden, and 5,800 (11.17%) were on disability pension at cohort entry. The mean (SD) age was 26.6 (11.0) years at first CUD diagnosis. The individuals were followed up for 8.69 years (SD 6.39) on average. The first recorded CUD diagnosis came more frequently from outpatient treatment (36,500; 70.26%) than inpatient treatment (15,447; 29.74%). During the follow-up, 24,819 (47.78%) individuals were re-diagnosed with CUD. Of the whole cohort, 5,800 (11.17%) were on disability pension at baseline. The CUD cohort consisted mostly of individuals with 25,868 (49.80%) diagnosis of harmful use of cannabis (F12.1) followed by 12,811 (24.66%) individuals diagnosed with cannabis dependence (F12.2), 5,441 (10.47%) individuals diagnosed with cannabis intoxication (F12.0), 3,305 (6.36%) individuals diagnosed with unspecified cannabis use disorder (F12.9), and 2,921 (5.62%) diagnosed with cannabis-induced psychosis (F12.5) at baseline. Of the sample, 5,031 (9.68%) had a previous record of schizophrenia spectrum disorder, 2,233 (4.30%) bipolar disorder, 3,307 (6.37%) personality disorder, 9,739 (18.75%) ADHD diagnosis ever before cohort entry, and 12,614 (24.28%) had a previous record of other SUD diagnosis 1 year before cohort entry. For more detailed information, see Table 1 and Supplementary Table S2.

**Table 1.** Characteristics of the cannabis use disorder (CUD) sample (*n* = 51,947) Table 1. Long description.

Variable	Frequency	Percent
Year of cohort entry		
1989–1999	2,320	4.47%
2000–2009	10,853	20.89%
2010–2019	28,485	54.83%
2020–2023	10,289	19.81%
Gender		
Female	12,671	24.39%
Male	39,276	75.61%
Born in Sweden		
Yes	41,670	80.22%
No	10,266	19.78%
CUD diagnosis at cohort entry		
F12.0 Acute cannabis intoxication	5,441	10.47%
F12.1 Harmful use of cannabis	25,868	49.80%
F12.2 Cannabis dependence	12,811	24.66%
F12.5 Cannabis-induced psychosis	2,921	5.62%
F12.9 Unspecified cannabis use disorder	3,305	6.36%
Other F12 diagnosis	1,601	3.08%
CUD diagnosis during the follow-up		
Yes	24,819	47.78%
No	27,128	52.22%
Other SUD diagnosis 1 year before cohort entry		
Yes	12,614	24.28%
No	39,333	75.72%
Psychiatric disorders ever before cohort entry		
Schizophrenia-spectrum disorder	5,031	9.68%
Bipolar disorder	2,233	4.30%
Personality disorder	3,307	6.37%
Attention deficit hyperactivity disorder	9,739	18.75%
Care setting at cohort entry		
Inpatient	15,447	29.74%
Outpatient	36,500	70.26%
Disability pension at cohort entry		
No	46,146	88.83%
Yes	5,800	11.17%

Abbreviation: SUD, substance use disorder.

### Risk of hospital admission related to SUD

After cohort entry, 22,598 (43.5% of the CUD cohort) individuals were recorded with a subsequent hospitalisation related to SUD. At group level, ADHD – medication (aHR: 0.85; 95% CI: 0.81–0.88) and SUD medication use (vs. no use) (aHR: 0.97; 95% CI: 0.93–0.99) were associated with decreased risk of hospitalisation related to SUD. In contrast any antipsychotic use was associated with increased risk of hospital admission related to SUD (aHR: 1.12; 95% CI: 1.09–1.15) as was any antidepressant use (aHR: 1.09; 95% CI: 1.06–1.11) and benzodiazepine use (aHR: 1.14; 95% CI: 1.11–1.17), while mood stabiliser use (aHR: 0.97; 95% CI: 0.93–1.01) did not reach statistical significance. Among the specific medication categories, use of clozapine (aHR: 0.47; 95% CI: 0.31–0.73), lisdexamfetamine (aHR: 0.74; 95% CI: 0.69–0.79), naltrexone (aHR: 0.79; 95% CI: 0.71–0.88), ADHD polytherapy (aHR: 0.85; 95% CI: 0.75–0.96), methylphenidate (aHR: 0.90; 95% CI: 0.86–0.94) and buprenorphine (aHR: 0.92; 95% CI: 0.88–0.96) were associated with a decreased risk of hospitalisation related to SUD. Results for all the specific pharmacotherapies, number of users, events and person-years are listed in Figure 1.

**Figure 1.**
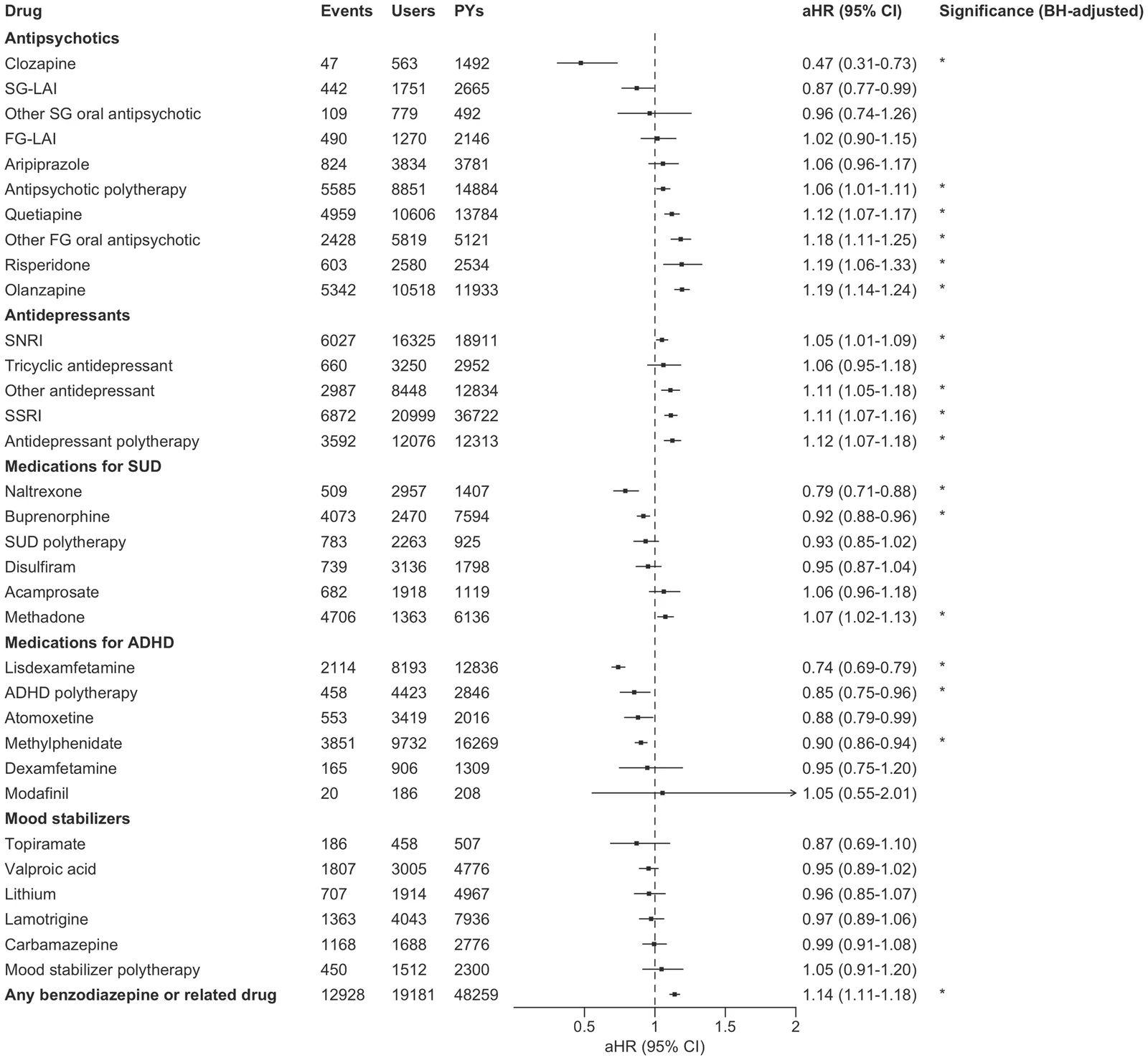
Association between psychiatric medication use and hospitalisation related to substance use disorder (SUD). *Note*: ADHD, attention deficit hyperactivity disorder; FG, first generation; FG-LAI, first-generation antipsychotic long-acting injection; SG, second generation; SG-LAI, second-generation antipsychotic long-acting injection; SNRI, serotonin and norepinephrine reuptake inhibitor; SSRI, selective serotonin reuptake inhibitor; SUD, substance use disorder; PYs, person-years; aHR, adjusted hazard ratios. *Asterisk* (*) denotes FDR-adjusted *p* < 0.05 (Benjamini–Hochberg correction). Figure 1. long description.

### Risk of hospital admission related to CUD

After cohort entry, 9,719 (18.7%) were recorded with a subsequent hospitalisation related to CUD. Among the specific medication categories, use of SG-LAI (aHR: 0.52; 95% CI: 0.36–0.56) and lisdexamfetamine (aHR: 0.56; 95% CI: 0.40–0.79) were associated with a decreased risk of hospitalisation. Results for all the specific pharmacotherapies, number of users, events, and person-years are listed in Figure 2.

**Figure 2.**
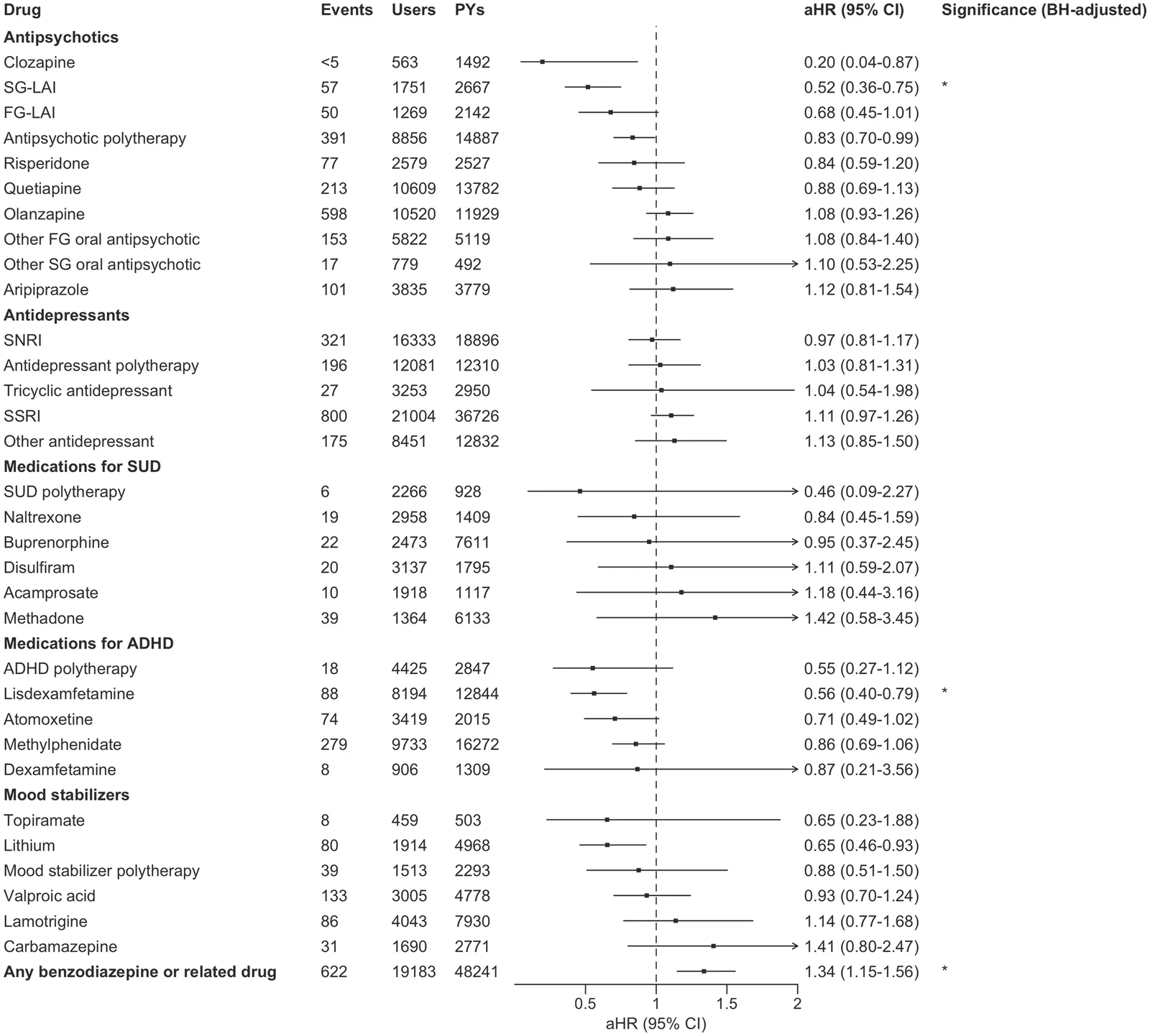
Association between psychiatric medication use and hospitalisation related to cannabis use disorder (CUD). *Note*: ADHD, attention deficit hyperactivity disorder; FG, first generation; FG-LAI, first-generation antipsychotic long-acting injection; SG, second generation; SG-LAI, second-generation antipsychotic long-acting injection; SNRI, serotonin and norepinephrine reuptake inhibitor; SSRI, selective serotonin reuptake inhibitor; PYs, person-years; aHR, adjusted hazard ratios. *Asterisk* (*) denotes FDR-adjusted *p* < 0.05 (Benjamini–Hochberg correction). Figure 2. long description.

### Risk of hospital admission related to any psychiatric disorder

After cohort entry, 27,360 (52.7%) individuals were recorded with a subsequent hospitalisation related to any psychiatric disorder. Among the specific medication categories, use of clozapine (aHR: 0.77; 95% CI: 0.68–0.87), lisdexamfetamine (aHR: 0.79; 95% CI: 0.76–0.83), naltrexone (aHR: 0.86; 95% CI: 0.79–0.93), buprenorphine (aHR: 0.88; 95% CI: 0.84–0.92), SG-LAI (aHR: 0.89; 95% CI: 0.83–0.95), methylphenidate (aHR: 0.94; 95% CI: 0.91–0.98), and ADHD polytherapy (aHR: 0.94; 95% CI: 0.91–0.98) were associated with a decreased risk of hospitalisation related to any psychiatric disorder. Results for all the specific pharmacotherapies, number of users, events, and person-years are listed in Figure 3.

**Figure 3.**
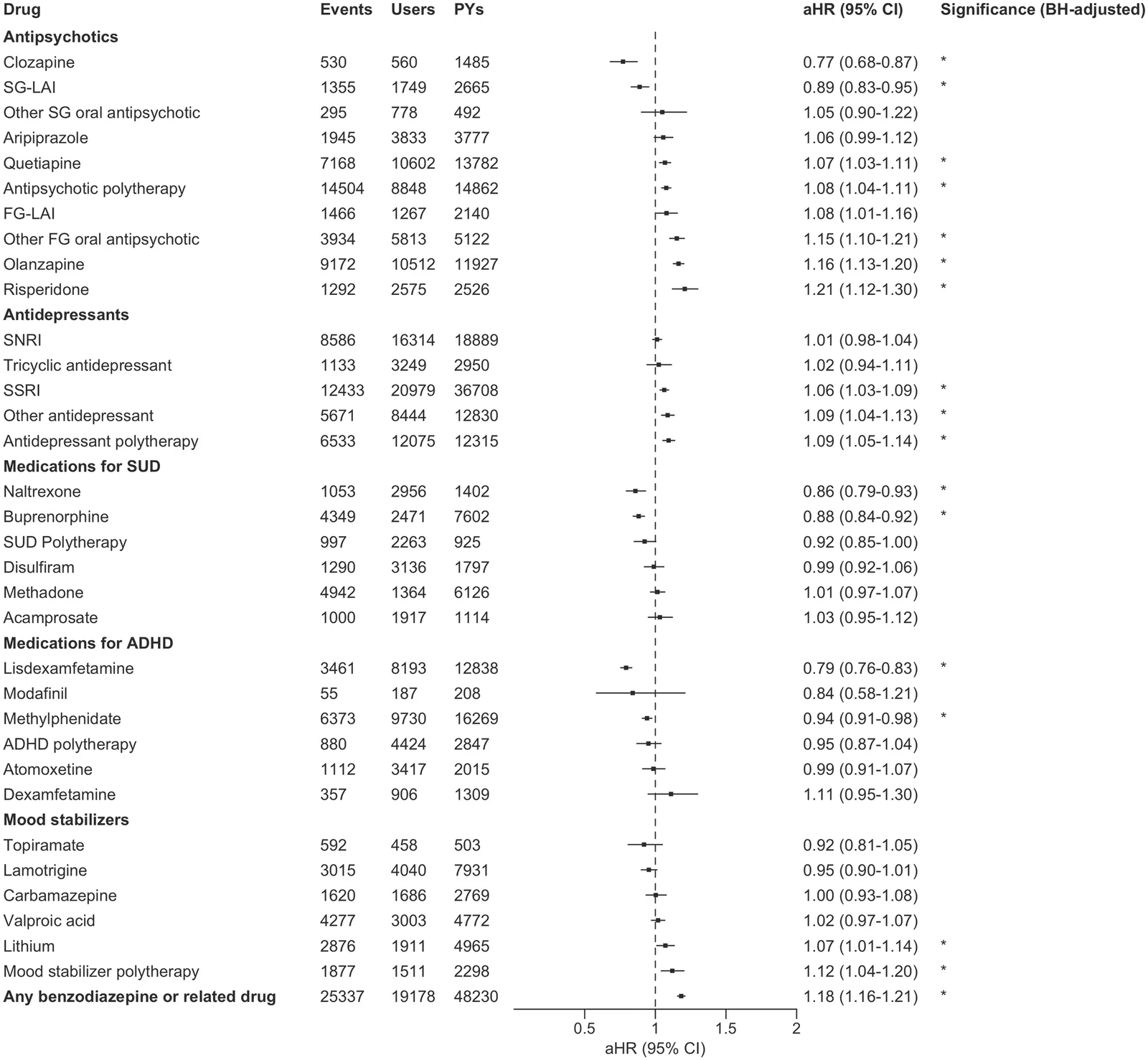
Association between psychiatric medication use and hospitalisation related to any psychiatric disorder. *Note*: ADHD, attention deficit hyperactivity disorder; FG, first generation; FG-LAI, first-generation antipsychotic long-acting injection; SG, second generation; SG-LAI, second-generation antipsychotic long-acting injection; SNRI, serotonin and norepinephrine reuptake inhibitor; SSRI, selective serotonin reuptake inhibitor; SUD, substance use disorder; PYs, person-years; aHR, adjusted hazard ratios. *Asterisk* (*) denotes FDR-adjusted *p* < 0.05 (Benjamini–Hochberg correction). Figure 3. long description.

## Discussion

This nationwide observational register-linkage study confirms the main conclusions of previous Cochrane reviews regarding the limited effectiveness of most pharmacotherapies in the treatment of CUD. Our findings indicate that most antipsychotics, antidepressants, benzodiazepines, and mood stabilisers were not associated with reduced risk of hospitalisations related to SUD, CUD, or other psychiatric disorders among individuals diagnosed with CUD. In contrast, the use of most antipsychotics and antidepressants was generally associated with an increased risk of SUD and any psychiatric hospitalisations in within-individual models, suggesting that during periods of medication use, these individuals experience worse outcomes. However, we observed possible benefits associated with medications for SUD and ADHD, clozapine, and second-generation LAIs, which likely reflect treatment effects in comorbid psychiatric conditions. Our findings support a cautious approach to prescribing in CUD unless addressing another primary indication, such as a comorbid psychotic disorder.

Despite the fact that most pharmacotherapies were not associated with a lower risk of hospitalisation, some specific medications were associated with a decreased risk. For example, lisdexamfetamine was associated with a decreased risk of hospitalisations related to SUD, CUD, or any psychiatric disorder, and methylphenidate with a decreased risk of hospitalisations related to SUD or any psychiatric disorder. Findings concerning stimulant medications align with the limited number of studies where a reduction in cannabis use, but not abstinence, among individuals with comorbid CUD and ADHD receiving stimulant medication treatment has been reported [18]. Naltrexone and buprenorphine were also associated with a decreased risk of hospitalisations related to SUD and any psychiatric disorders, and these findings remained consistent for SUD-related hospitalisation in the sensitivity analysis that excluded individuals with an SUD diagnosis during the year before cohort entry. Regarding medications for SUD, no trials have specifically demonstrated that opioid substitution therapy improves outcomes related to CUD. The existing evidence primarily concerns opioid use disorder (OUD), where better long-term outcomes, including improved treatment retention and reduced mortality, are established [19]. Preliminary evidence for naltrexone in the treatment of CUD remains inconclusive [6, 20], yet its real-world effectiveness has been demonstrated for alcohol use disorder (AUD) within this same dataset [21]. Moreover, naltrexone has been suggested as a potential option for the treatment of amphetamine use disorder, indicating possible broader applicability across substance use disorders [22–24].

Furthermore, clozapine use was associated with a reduced risk of hospitalisations related to SUD and any psychiatric disorder, while SG-LAI use was associated with a reduced risk of hospitalisations related to CUD and any psychiatric disorder. Considering that clozapine is indicated for treatment-resistant schizophrenia and SG-LAIs are primarily prescribed for psychotic disorders, these findings likely reflect their strong effectiveness in managing psychotic disorders, as demonstrated in our recent studies on cannabis-induced psychosis [25] and first-episode psychosis with comorbid CUD [26]. Nevertheless, clozapine may also exert beneficial effects on reducing cannabis use, as suggested by evidence of greater substance-use abstinence among individuals treated with clozapine compared to other antipsychotics [27–30].

As these medications are indicated solely for psychotic disorders, OUD, AUD, and ADHD, these findings may reflect beneficial effects on comorbidities that also contribute to poorer outcomes in individuals with CUD. Furthermore, individuals who adhere to the rigorous monitoring required in clozapine treatment may demonstrate superior clinical outcomes. Similarly, patients with CUD who receive lisdexamfetamine or methylphenidate may represent a highly selected subgroup with comorbid ADHD who are actively engaged in care and exhibit different patterns of treatment adherence, potentially limiting the generalisability of these findings outside the ADHD population. Although clozapine, SG-LAIs, buprenorphine, naltrexone, methylphenidate, and lisdexamfetamine demonstrated real-world effectiveness even after false-discovery rate correction, this study remains exploratory and observational in nature. Therefore, any positive findings should not be interpreted as clinical recommendations but rather as guidance for future RCTs on the treatment of CUD. However, our findings highlight the importance of addressing comorbidity and suggest that future trials should also explore whether pharmacotherapy for psychotic disorders, ADHD, or other SUDs can improve outcomes in CUD.

Beyond medication-related aspects, functional outcomes also warrant attention. More than 11% of this cohort were receiving disability pension at cohort entry, which exceeds the rates reported in our recent studies of individuals with cannabis-induced psychosis (6.94%) [25] and first-episode psychosis with comorbid CUD (9.73%) [26]. This finding suggests that CUD diagnosis may represent a negative prognostic marker for work-life trajectories, as this rate is higher than among individuals diagnosed with psychotic disorders. These results further emphasise the importance of early identification and targeted interventions for CUD to mitigate functional decline in these individuals.

Our findings should be interpreted in light of several important limitations. The large number of medication classes, individual agents, and outcomes also raises the possibility of chance findings despite FDR correction, reinforcing the exploratory nature of our work. This study identified individuals based on their first recorded CUD diagnosis, followed them over time, and focused on hospitalisation as an outcome. We lacked information on the continuation of cannabis use beyond register-based CUD diagnoses, although the persistence of CUD diagnoses over time (47.8% were re-diagnosed by the end of follow-up) suggests ongoing cannabis-related problems. There was substantial psychiatric comorbidity at baseline, which likely explains the positive associations observed for stimulant medications, certain antipsychotics, and medications for SUD. Therefore, these real-world effectiveness estimates are better explained by benefits for comorbid conditions than by a CUD-specific impact. Only 18.5% of individuals experienced a hospital admission related to CUD during follow-up. Consequently, models assessing CUD-related hospitalisation may be underpowered to detect potential associations for some medication groups. Moreover, our findings are likely more generalisable to the most severe phenotypes of CUD rather than the full spectrum of cases. Given that the majority of individuals received their CUD diagnosis in an outpatient setting at cohort entry (70.3%), our models examining medication use and risk of hospitalisation may not capture all possible treatment effects observed in outpatient care, many of which could lead to meaningful improvements in quality of life and prognosis.

However, there are also major strengths that outline the importance of our research. The nationwide register-based design and having information on all Swedish residents provides exceptional generalisability, and the use of within-person analyses eliminates the effect of familial and genetic confounders. This study is the first to examine the long-term outcomes of different psychiatric medications after first diagnosis of CUD. Drawing on data from over 51,000 individuals, our findings highlight the importance of exercising general caution in the prescription of widely used psychiatric pharmacotherapies in preventing hospitalisation in individuals with CUD. Importantly, sensitivity analyses for the primary outcome generally followed a similar pattern for SUD-related outcomes, reinforcing the robustness of our results. These novel findings are likely to contribute to the development of future clinical recommendations, reinforce the conclusions of Cochrane reviews, and inform the design of RCTs focusing on the treatment of comorbid psychiatric disorders and CUD.

## Supporting information

10.1192/j.eurpsy.2026.12250.sm001 Mustonen et al. supplementary material Mustonen et al. supplementary material

## Data Availability

The data used in this study cannot be made publicly available due to privacy regulations. According to the General Data Protection Regulation, the Swedish law SFS 2018:218, the Swedish Data Protection Act, the Swedish Ethical Review Act, and the Public Access to Information and Secrecy Act, these types of sensitive data can only be made available for specific purposes, including research, that meet the criteria for access to this sort of sensitive and confidential data as determined by a legal review. Readers may contact Professor Ellenor Mittendorfer-Rutz (ellenor.mittendorfer-rutz@ki.se) regarding the data.

## Supplementary material

The supplementary material for this article can be found at http://doi.org/10.1192/j.eurpsy.2026.12250.

## Long descriptions

### Table 1. Long description

The table is organized into three columns: Variable, Frequency, and Percent.

* Year of cohort entry: 1989 to 1999 (2,320, 4.47 percent); 2000 to 2009 (10,853, 20.89 percent); 2010 to 2019 (28,485, 54.83 percent); 2020 to 2023 (10,289, 19.81 percent).

* Gender: Female (12,671, 24.39 percent); Male (39,276, 75.61 percent).

* Born in Sweden: Yes (41,670, 80.22 percent); No (10,266, 19.78 percent).

* C U D diagnosis at cohort entry: F 1 2 dot 0 Acute cannabis intoxication (5,441, 10.47 percent); F 1 2 dot 1 Harmful use of cannabis (25,868, 49.80 percent); F 1 2 dot 2 Cannabis dependence (12,811, 24.66 percent); F 1 2 dot 5 Cannabis-induced psychosis (2,921, 5.62 percent); F 1 2 dot 9 Unspecified cannabis use disorder (3,305, 6.36 percent); Other F 1 2 diagnosis (1,601, 3.08 percent).

* C U D diagnosis during the follow-up: Yes (24,819, 47.78 percent); No (27,128, 52.22 percent).

* Other S U D diagnosis 1 year before cohort entry: Yes (12,614, 24.28 percent); No (39,333, 75.72 percent).

* Psychiatric disorders ever before cohort entry: Schizophrenia-spectrum disorder (5,031, 9.68 percent); Bipolar disorder (2,233, 4.30 percent); Personality disorder (3,307, 6.37 percent); Attention deficit hyperactivity disorder (9,739, 18.75 percent).

* Care setting at cohort entry: Inpatient (15,447, 29.74 percent); Outpatient (36,500, 70.26 percent).

* Disability pension at cohort entry: No (46,146, 88.83 percent); Yes (5,800, 11.17 percent).

Navigate back to Table 1..

### Figure 1. Long description

A forest plot table with columns for Drug, Events, Users, P Y s, a visual hazard ratio plot, and a H R 95 percent C I. A vertical dashed line at 1.0 indicates no difference in risk.

* Antipsychotics: Clozapine has the lowest risk at 0.47. S G L A I is 0.87. Other S G oral, F G L A I, Aripiprazole, and Antipsychotic polytherapy range from 0.96 to 1.06. Quetiapine 1.12, Other F G oral 1.18, Risperidone 1.19, and Olanzapine 1.19 show significantly increased risk.

* Antidepressants: All show slightly increased risk. S N R I 1.05, Tricyclic 1.06, Other antidepressant 1.11, S S R I 1.11, and Antidepressant polytherapy 1.12.

* Medications for S U D: Naltrexone 0.79 and Buprenorphine 0.92 show significantly reduced risk. S U D polytherapy 0.93 and Disulfiram 0.95 are below 1. Acamprosate 1.06 and Methadone 1.07 are above 1.

* Medications for A D H D: All show reduced risk except Modafinil. Lisdexamfetamine 0.74, A D H D polytherapy 0.85, Atomoxetine 0.88, and Methylphenidate 0.90 are significant. Dexamfetamine is 0.95 and Modafinil is 1.05.

* Mood stabilizers: Topiramate 0.87, Valproic acid 0.95, Lithium 0.96, Lamotrigine 0.97, and Carbamazepine 0.99 all have C I crossing 1.0. Mood stabilizer polytherapy is 1.05.

* Any benzodiazepine or related drug: Shows a significantly increased risk at 1.14.

Navigate back to Figure 1..

### Figure 2. Long description

A forest plot table with columns for Drug, Events, Users, P Y s, a graphical representation of a H R (95 percent C I), and Significance (B H-adjusted). A vertical dashed line at 1.0 indicates the null effect.

* Antipsychotics: Clozapine shows the lowest risk at 0.20. S G-L A I (0.52) and Antipsychotic polytherapy (0.83) also show significantly reduced risk. Olanzapine, Other F G oral, Other S G oral, and Aripiprazole are to the right of the null line.

* Antidepressants: S N R I (0.97), Antidepressant polytherapy (1.03), Tricyclic (1.04), S S R I (1.11), and Other (1.13) all have confidence intervals crossing 1.0.

* Medications for S U D: S U D polytherapy (0.46), Naltrexone (0.84), and Buprenorphine (0.95) are below 1.0, while Disulfiram (1.11), Acamprosate (1.18), and Methadone (1.42) are above, though all intervals cross 1.0.

* Medications for A D H D: Lisdexamfetamine shows a significant reduction (0.56). A D H D polytherapy (0.55), Atomoxetine (0.71), Methylphenidate (0.86), and Dexamfetamine (0.87) are also below 1.0.

* Mood stabilizers: Lithium (0.65) shows a significant reduction. Topiramate (0.65), Mood stabilizer polytherapy (0.88), and Valproic acid (0.93) are below 1.0, while Lamotrigine (1.14) and Carbamazepine (1.41) are above.

* Any benzodiazepine or related drug: Shows a significant increase in risk at 1.34 (1.15-1.56).

Navigate back to Figure 2..

### Figure 3. Long description

The forest plot is organized into five main categories: Antipsychotics, Antidepressants, Medications for S U D, Medications for A D H D, and Mood stabilizers. The x-axis represents the adjusted hazard ratio (a H R) on a scale from 0.5 to 2, with a vertical dashed line at 1.0 indicating no difference in risk.

* Antipsychotics: Clozapine (a H R 0.77) and S G L A I (a H R 0.89) are significantly below 1.0. Risperidone (a H R 1.21) and Olanzapine (a H R 1.16) show significantly increased risk.

* Antidepressants: S S R I (a H R 1.06), Other antidepressant (a H R 1.09), and Antidepressant polytherapy (a H R 1.09) show significantly increased risk. S N R I and Tricyclic antidepressants cross the line of unity.

* Medications for S U D: Naltrexone (a H R 0.86) and Buprenorphine (a H R 0.88) show significantly reduced risk. Others like Methadone and Acamprosate are near 1.0.

* Medications for A D H D: Lisdexamfetamine (a H R 0.79) and Methylphenidate (a H R 0.94) show significantly reduced risk. Dexamfetamine (a H R 1.11) shows a non-significant trend toward increased risk.

* Mood stabilizers: Lithium (a H R 1.07) and Mood stabilizer polytherapy (a H R 1.12) show significantly increased risk. Topiramate and Lamotrigine trend below 1.0 but are not statistically significant.

* Any benzodiazepine or related drug: Shows a significantly increased risk with an a H R of 1.18.

Asterisks indicate B H-adjusted significance for several medications across all categories except Mood stabilizers where only Lithium and polytherapy are significant.

Navigate back to Figure 3..
