# Supplementary material for: Real-world effectiveness of psychiatric pharmacotherapies in reducing the risk of substance use- and psychiatric hospitalisations in cannabis use disorder – An exploratory nationwide register-based study
Source: Eur Psychiatry. 2026 Aug 12;69(1):e89. doi: 10.1192/j.eurpsy.2026.12250 (PMC13735834; doi:10.1192/j.eurpsy.2026.12250)
Supplement: Supplementary file 1 [file S0924933826122500sup001.docx]

**METHODS**

**Exposure variables**

Medication data were gathered from the PDR from July 2005 to December 2024 and were categorised into different pharmacotherapies based on Anatomical Therapeutic Chemical (ATC) classification codes. These pharmacotherapies included antipsychotics (N05A, excluding N05AN01 (lithium)) that were categorised into oral formulations of risperidone, aripiprazole, olanzapine, clozapine, and quetiapine. We pooled some medications for clinically relevant categories and to provide sufficient sample sizes. Use of other oral first – generation antipsychotic medications as ‘other FG oral antipsychotic monotherapy’, use of other second-generation antipsychotic medications as ‘other SG oral antipsychotic monotherapy’, use of two or more concurrent antipsychotics as ‘antipsychotic polytherapy’, all long-acting injectable (LAI) formulations of first-generation antipsychotics as ‘first-generation LAI’ (FG-LAI) and all LAI formulations of second-generation antipsychotics as ‘second-generation LAI’ (SG-LAI). Use of antidepressants (N06A) were categorised as ‘Tricyclic antidepressants’, ‘SSRIs’, ‘SNRIs’, ‘other antidepressants’ and use of two or more concurrent antidepressants as ‘AD polytherapy’. Mood stabilizers (N03AF01, N03AG01, N03AX09, N05AN01) were categorised as carbamazepine, valproic acid, lamotrigine, topiramate, lithium and use of two or more concurrent antidepressants as ‘Mood stabilizer polytherapy’. Medications (N06BA) for attention-deficit hyperactivity disorder (ADHD) were categorised as dexamfetamine, methylphenidate, modafinil, atomoxetine, lisdexamphetamine and use of two or more concurrent ADHD medications as ‘ADHD polytherapy’. Medications (N07BB, N07BC) for substance use disorders (SUD) were categorised as disulfiram, acamprosate, naltrexone, buprenorphine, methadone and use of two or more concurrent SUD medications as ‘SUD polytherapy’. Use of benzodiazepines and related drugs (N05BA, N05CD, N05CF) were categorised as ‘Any benzodiazepine and related drug’.

**RESULTS (Sensitivity Analysis)**

**Supplement figure 1.** Association between psychiatric medication use and hospitalisation related to substance use disorder (SUD) in a cohort excluding individuals with an SUD diagnosis during the year prior to cohort entry.

**Footnote:** ADHD = Attention deficit hyperactivity disorder, FG = first-generation, FG-LAI = first-generation antipsychotic long-acting injection, SG = second generation, SG-LAI = second-generation antipsychotic long-acting injection. SNRI = serotonin and norepinephrine reuptake inhibitor, SSRI = selective serotonin reuptake inhibitor, SUD = substance use disorder, aHR = adjusted hazard ratios. Asterisk (*) denotes FDR-adjusted p < 0.05 (Benjamini–Hochberg correction).

**Supplement figure 2.** Association between psychiatric medication use and hospitalisation related to substance use disorder (SUD) in between-individual models.

**Footnote:** ADHD = Attention deficit hyperactivity disorder, FG = first-generation, FG-LAI = first-generation antipsychotic long-acting injection, SG = second generation, SG-LAI = second-generation antipsychotic long-acting injection. SNRI = serotonin and norepinephrine reuptake inhibitor, SSRI = selective serotonin reuptake inhibitor, SUD = substance use disorder, aHR = adjusted hazard ratios. Asterisk (*) denotes FDR-adjusted p < 0.05 (Benjamini–Hochberg correction).

**Supplement Table 1. Covariate definitions.**

| Covariate | Definition | Register source | Models utilizing the covariate |
| --- | --- | --- | --- |
| Order of treatments | Temporal order of treatments, continuously updated in the models and categorized as first, second, third and >third | PDR | W, B |
| Time since cohort entry | Continuously updated in the models, categorized as 0-1, 1-3 and >3 years |  | W |
| Antipsychotics | Continuously updated in the models, ATC N05A, excluding N05AN01 | PDR | W, B |
| Antidepressants | Continuously updated in the models, ATC N06A | PDR | W, B |
| Benzodiazepines and related drugs | Continuously updated in the models, ATC N05BA, N05CD, N05CF | PDR | W, B |
| ADHD medications | Continuously updated in the models , ATC N06BA | PDR | W, B |
| SUD medications | Continuously updated in the models, ATC N07BB medications used in alcohol dependence, N07BC medications used in opioid use disorder | PDR | W, B |
| Mood stabilizers | Continuously updated in the models, ATC N03AF01, N03AG01, N03AX09, N05AN01 | PDR | W, B |
| Age | Age at cohort entry | LISA | B |
| Gender | Man vs. woman | LISA | B |
| Disability pension | Continuously updated in the models, no vs. yes after the first granted decision. | MiDAS | B |
| Number of previous hospitalizations due to cannabis use disorder | Continuously updated in the models and categorized as 0, 1, 2-3 vs. >3 | NPR | B |
| ADHD | Continuously updated in the models with status ”no” until the first diagnosis ICD-10 F90 occurred and ”yes” thereafter. | NPR | B |
| Personality disorders | Continuously updated in the models with status ”no” until the first diagnosis ICD-10 F60-F69 occurred and ”yes” thereafter. | NPR | B |
| Epilepsy | Continuously updated in the models with status ”no” until the first diagnosis ICD-10 G40 occurred and ”yes” thereafter. | NPR | B |
| Diabetes | Continuously updated in the models with status ”no” until the first diagnosis ICD-10 E08–E13 occurred and ”yes” thereafter. | NPR | B |
| Cancer | Continuously updated in the models with status ”no” until the first diagnosis ICD-10 C00-C97 occurred and ”yes” thereafter. | NPR | B |
| Cardiovascular disease | Continuously updated in the models with status ”no” until the first diagnosis ICD-10 I00–I99, occurred and ”yes” thereafter. | NPR | B |
| Brain injury | Continuously updated in the models with status ”no” until the first diagnosis ICD-10 S06 occurred and ”yes” thereafter. | NPR | B |
| Previous suicide attempt | Continuously updated in the models with status ”no” until the first diagnosis ICD-10 X60-X84, Y10-Y34 occurred and ”yes” thereafter. | NPR | B |
| Previous accidental overdose | Continuously updated in the models with status ”no” until the first diagnosis ICD-10 X40-X49 occurred and ”yes” thereafter. | NPR | B |
| PDR: Prescribed Drug Register, LISA: The Longitudinal Integration Database for Health Insurance and Labor Market Studies, NPR: National Patient register, MiDAS: the Microdata for Analyses of Social Insurance.  W=within-individual models, B=between-individual models. | | | |

| **Supplement table 2. Characteristics of the cannabis use disorder (CUD) sample (n= 51 947).** | | |
| --- | --- | --- |
| **Variable** | **Frequency** | **Percent** |
| **SUD diagnosis 1 year before cohort entry*** |  |  |
| F10 Alcohol use disorder | 6059 | 11.66 % |
| F11 Opioid use disorder | 2845 | 5.48 % |
| F13 Sedative use disorder | 3547 | 6.83 % |
| F15 Stimulant use disorder | 4029 | 7.76 % |
| F19 Polysubstance use disorder | 4904 | 9.44 % |
| Other SUDs (F14, F16, F17, F18) | 1905 | 3.67 % |
| **SUD diagnosis ever before cohort entry*** |  |  |
| F10 Alcohol use disorder | 13211 | 25.45 % |
| F11 Opioid use disorder | 5310 | 10.22 % |
| F13 Sedative use disorder | 5865 | 11.29 % |
| F15 Stimulant use disorder | 5990 | 11.53 % |
| F19 Polysubstance use disorder | 11610 | 22.35 % |
| Other SUDs (F14, F16, F17, F18) | 3001 | 5.78 % |
| **SUD diagnosis during follow-up** |  |  |
| F10 Alcohol use disorder | 6574 | 12.66 % |
| F11 Opioid use disorder | 4523 | 8.71 % |
| F13 Sedative use disorder | 4688 | 9.02 % |
| F15 Stimulant use disorder | 4068 | 7.83 % |
| F19 Polysubstance use disorder | 12134 | 23.36 % |
| Other SUDs (F14, F16, F17, F18) | 3830 | 7.37 % |

*Before first diagnosis of CUD. SUD = Subtance use disorder
